# Development and Implementation of Preoperative Optimization for High-Risk Patients With Abdominal Wall Hernia

**DOI:** 10.1001/jamanetworkopen.2021.6836

**Published:** 2021-05-12

**Authors:** Ryan Howard, Lia Delaney, Amy M. Kilbourne, Kelley M. Kidwell, Shawna Smith, Michael Englesbe, Justin Dimick, Dana Telem

**Affiliations:** 1 Division of Minimally Invasive Surgery, Department of Surgery, University of Michigan, Ann Arbor; 2 University of Michigan Medical School, Ann Arbor; 3 Health Services Research and Development, Office of Research and Development, US Department of Veterans Affairs, Washington, DC; 4 University of Michigan School of Public Health, Ann Arbor

## Abstract

**Importance:**

Real-world surgical practice often lags behind the best scientific evidence. For example, although optimizing comorbidities such as smoking and morbid obesity before ventral and incisional hernia repair improves outcomes, as many as 25% of these patients have a high-risk characteristic at the time of surgery. Implementation strategies may effectively increase use of evidence-based practice.

**Objective:**

To describe current trends in preoperative optimization among patients undergoing ventral hernia repair, identify barriers to optimization, develop interventions to address these barriers, and then pilot these interventions.

**Design, Setting, and Participants:**

This quality improvement study used a retrospective medical record review to identify hospital-level trends in preoperative optimization among patients undergoing ventral and incisional hernia repair. Semistructured interviews with 21 practicing surgeons were conducted to elicit barriers to optimizing high-risk patients before surgery. Next, a task force of experts was convened to develop pragmatic interventions to increase surgeon use of preoperative optimization. Finally, these interventions were piloted at 2 sites to assess acceptability and feasibility. This study was performed from January 1, 2014, to December 31, 2019.

**Main Outcomes and Measures:**

The main outcome was rate of referrals for preoperative patient optimization at the 2 pilot sites.

**Results:**

Among 23 000 patients undergoing ventral hernia repair, the mean (SD) age was 53.9 (14.3) years, and 12 315 (53.5%) were men. Of these, 8786 patients (38.2%) had at least 1 high-risk characteristic at the time of surgery, including 7683 with 1, 1079 with 2, and 24 with 3. At the hospital level, the mean proportion of patients with at least 1 of 3 high-risk characteristics at the time of surgery was 38.2% (95% CI, 38.1%-38.3%). This proportion varied widely from 21.5% (95% CI, 17.6%-25.5%) to 52.8% (95% CI, 43.9%-61.8%) across hospitals. Interviews with surgeons identified 3 major barriers to improving this practice: lost financial opportunity by not offering a patient an operation, lack of surgeon awareness of available resources for optimization, and organizational barriers. A task force therefore developed 3 interventions: a financial incentive to optimize high-risk patients, an educational intervention to make surgeons aware of available optimization resources, and on-site facilitation. These strategies were piloted at 2 sites where preoperative risk optimization referrals increased 860%.

**Conclusions and Relevance:**

This study demonstrates a stepwise process of identifying a practice gap, eliciting barriers that contribute to this gap, using expert consensus and local resources to develop strategies to address these barriers, and piloting these strategies. This implementation strategy can be adopted to diverse settings given that it relies on developing and implementing strategies based on local practice patterns.

## Key Points

**Question:**

Is a stepwise implementation intervention associated with increased use of preoperative optimization by surgeons for patients with abdominal wall hernia?

**Findings:**

In this quality improvement study including 23 000 patients, preoperative optimization for high-risk patients with abdominal wall hernia varied from 20% to 53% across 73 hospitals owing to barriers that included lack of surgeon knowledge and financial concerns. An intervention involving on-site education and financial incentivization increased referral for preoperative optimization by 860%.

**Meaning:**

Implementation strategies that address real-world barriers may be an effective method to increase surgeon use of evidence-based practice.

## Teaser

This quality improvement study describes current trends in preoperative patient optimization, identifies barriers to optimization, develops interventions to address these barriers, and then pilots these interventions among high-risk patients undergoing ventral and incisional hernia repair.

## Introduction

Surgical care in the United States is expensive and is associated with substantial mortality rates.^1,2^ Improvement efforts have traditionally focused on the procedure itself and perioperative care.^3^ Much less attention has been given to the critical decision of when to operate and on whom, which is inextricably linked to patient outcomes. For instance, patients with physical deconditioning, excessive weight, active substance abuse, or poorly controlled diabetes have markedly higher rates of surgical complications.^4^ In fact, these factors are considered relative contraindications to elective procedures such as joint replacement and hernia surgery.^5,6,7,8,9,10,11,12^ Data suggest that delaying surgery and optimizing or improving patients’ risk factors preoperatively can reduce surgical complication rates by as much as 40%.^13,14,15^

Surgeons often fail to adopt scientific evidence in their decision-making about which patients are likely to benefit from surgery.^16,17^ Abdominal wall hernia repair is an exemplary condition in which inattention to preoperative optimization is associated with increased complications and costs after surgery. Abdominal wall hernia repair is one of the most common operations performed in the US, with more than 500 000 repairs performed annually.^18^ Despite the overwhelming evidence of the benefits of preoperative optimization and delaying elective abdominal wall hernia repair until patient health and risk factors can be optimized, a significant practice gap persists with regard to best practices for patient optimization before hernia repair. In a large population-based study,^4^ as many as 25% of patients undergoing elective abdominal wall hernia repair did not undergo optimization before surgery. This finding was associated with increased short-term morbidity and an additional US $60 million per annum in episode of care payments.

Within this context, we used an implementation science framework to inform a stepwise approach to implementing a multifaceted quality improvement intervention to bridge the gap between recommendation and adoption of best practices for preoperative optimization of patients with ventral hernia.^19^ Using the Theoretical Domains Framework,^20^ stakeholders (eg, surgeons) were interviewed to identify the most salient barriers and facilitators to practice change. Identified barriers were then mapped to theoretical domains and candidate techniques for evidence-based behavior change.^21^ This study explores the selection of evidence-based implementation strategies based on behavior change techniques and their initial piloting and assessment at 2 sites that perform abdominal wall hernia repair.

## Methods

**Figure 1.**
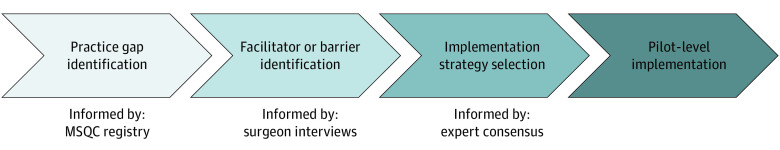
Overview of Intervention Development and Implementation Process MSQC indicates Michigan Surgical Quality Collaborative.

This study was designed to engage surgeons and institutional stakeholders in identifying barriers to preoperative optimization for patients with ventral hernia. Using this information, we sought to develop implementation strategies that would enable surgeons to use this evidence-based practice. We then evaluated referral for optimization after the intervention (Figure 1). This work was performed from January 1, 2014, to December 31, 2019.

This clinical quality improvement study was approved by the University of Michigan institutional review board and follows the Standards for Quality Improvement Reporting Excellence (SQUIRE) reporting guideline.^22^ The requirement for informed consent was waived by the institutional review board.

### Understanding the Practice Gap

A previous report by Howard et al^4^ found that 15% to 25% of patients with ventral hernia have high-risk characteristics at the time of surgery. The present study aims to understand hospital-level optimization of these risks. Specifically, to the extent that a given hospital has a high or low proportion of high-risk patients, opportunities for systematic quality improvement may exist. We therefore used data from the Michigan Surgical Quality Collaborative (MSQC) to describe site-level variation in preoperative optimization. The MSQC is a statewide clinical registry comprising 73 hospitals that prospectively collects data on perioperative processes of care and 30-day clinical outcomes after general surgical procedures. Data are reviewed and abstracted by trained nurse abstractors, and a sampling algorithm is used to minimize selection bias.^23^

Optimization was defined as the absence of a high-risk characteristic at the time of surgery. Variation in hospital-level optimization was described by the proportion of patients undergoing ventral hernia repair at each hospital who had a characteristic of tobacco use, morbid obesity, or unhealthy alcohol consumption at the time of surgery. Presence of a high-risk characteristic at the time of surgery was established by review of a patient’s complete medical record (eg, history and physical, daily progress notes, and discharge summary) by a trained clinical data abstractor. These data were obtained from January 1, 2014, to December 31, 2018.

### Identifying Evidence-Based Implementation Strategies

The MSQC has previously implemented statewide quality improvement campaigns using its collaborative structure, such as surgical site infection prevention and evidence-based opioid prescribing.^24,25,26^ Our goal was to understand the barriers and facilitators to preoperative optimization of patient risk that could inform implementation strategies, defined as highly specified, theory-based tools or methods designed to sustain quality improvement through implementation of effective practices.

To understand these barriers, we conducted qualitative interviews with a convenience sample of 21 practicing surgeons in Michigan who responded to a survey sent to 31 MSQC-participating surgeons. Surgeons were from community (n = 8) and academic (n = 13) hospitals and were required to be practicing surgeons who performed abdominal wall hernia repair. Interviews were conducted independently by telephone or in person. Some themes elicited from these interviews have been described previously; however, the present study specifically explores the elicited barriers in more detail as part of a stepwise implementation strategy.^27^

After these interviews, a multidisciplinary hernia task force was convened consisting of 25 experts in hernia surgery, pain management, anesthesia, quality improvement, data abstraction, and implementation. Participants were recruited based on existing networks within the MSQC with the purpose of improving the care of patients with hernia in Michigan. The study leads (M.E. and D.T.) shared the results of the qualitative interviews and held open discussion to solicit candidate strategies from participants during three 1-hour, in-person sessions. Given the goal of maximizing feasibility and acceptability, a pragmatic framework was used to identify candidate strategies that were most likely to be adopted within MSQC hospitals.^28^ Candidate strategies included those that had been used previously within the MSQC to motivate quality improvement initiatives and incorporated value-based reimbursement, pay-for-performance (P4P), case-based billing modifiers (eg, Modifier 22), in-person courses or seminars, and delivery of focused education and reminders to surgeons. Pragmatic strategies were given priority. Delphi and discrete choice models were not used. Three implementation strategies were ultimately selected based on identified barriers and facilitators, sources of behavior from linkage of theoretical framework domains to behavior change theories, results of prior successful strategies deployed through the MSQC, and established resources available within the MSQC.^25,29,30^

### Stepwise Implementation of Evidence-Based Interventions at a Pilot Level

Selected implementation strategies were piloted within 2 MSQC health care systems to evaluate feasibility and acceptability. As part of this pilot, surgeons at both sites were willing to participate in brief educational sessions to learn about the proposed interventions and resources. Pilot sites were also willing to host on-site facilitators who were added to increase early adoption of the implementation strategies developed as part of this study. The main outcome was the number of referrals placed for preoperative optimization at both pilot sites to measure any changes in optimization referral after implementation of this intervention.

### Statistical Analysis

A descriptive analysis was calculated for high-risk characteristics at the time of surgery across hospitals. Statistical analyses were performed using STATA, version 16.0 (StataCorp LLC).

## Results

### Hospital-Level Variation in Adherence to Preoperative Optimization

**Figure 2.**
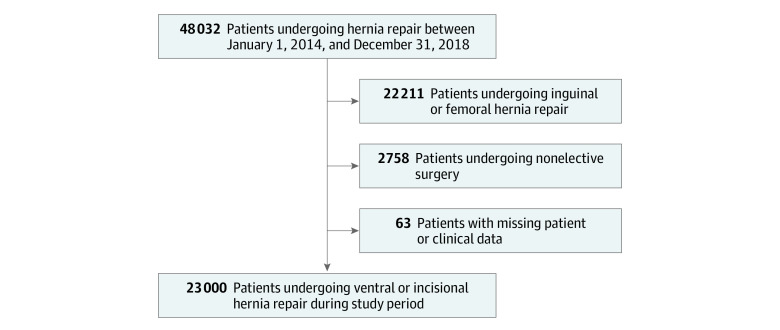
Cohort Selection

**Figure 3.**
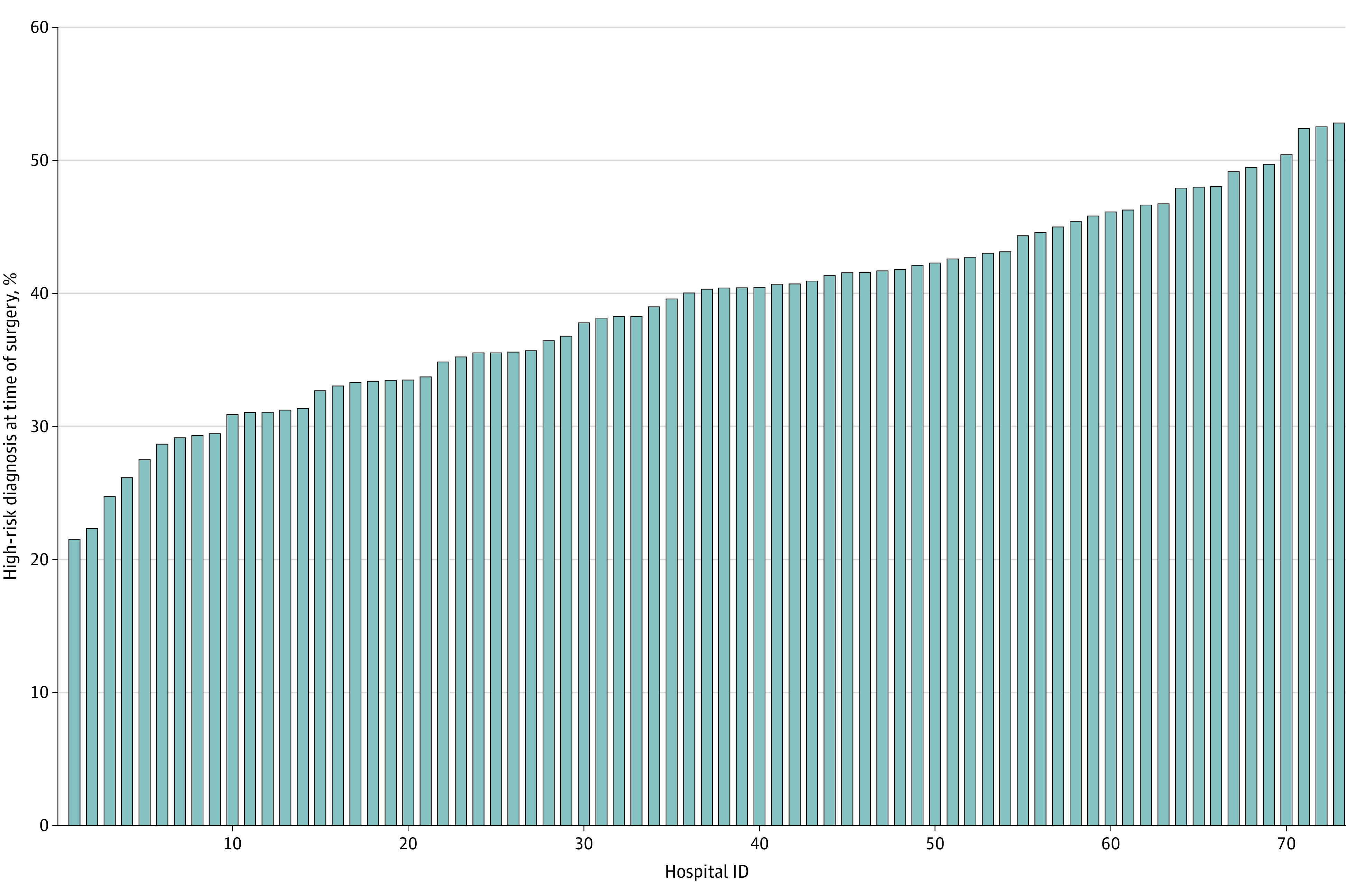
Aggregate Variation in Adherence to Preoperative Optimization Across Michigan Surgical Quality Collaborative Sites From 2014 to 2018 The population includes patients with active tobacco use, morbid obesity (body mass index [calculated as weight in kilograms divided by height in meters squared] ≥40), or unhealthy alcohol consumption at the time of surgery at the 73 study hospitals.

The patient selection for this study is presented in Figure 2. Among 23 000 patients undergoing ventral hernia repair during the study period, the mean (SD) age was 53.9 (14.3) years, 10 685 were women (46.5%), and 12 315 were men (53.5%). In terms of race/ethnicity, 19 504 patients (84.8%) were White, 2507 (10.9%) were Black, 145 (0.6%) were of another race/ethnicity, and 844 (3.7%) were of unknown race/ethnicity. At the hospital level, 8786 patients had at least 1 of 3 high-risk characteristics at the time of surgery for a mean proportion of 38.2% (95% CI, 38.1%-38.3%) (Figure 3). This proportion varied widely from 21.5% (95% CI, 17.6%-25.5%) to 52.8% (95% CI, 43.9%-61.8%) across hospitals. Of the 8786 patients, 7683 (87.4%) had 1 high-risk characteristic, 1079 (12.3%) had 2 high-risk characteristics, and 24 (0.3%) had all 3 high-risk characteristics at the time of surgery.

### Barriers to Preoperative Optimization

In a recent study, Vitous et al^27^ elicited 3 barriers to practice change that are explored herein in more detail as they apply to an overall implementation strategy (Table). The first theme centered on financial concerns about delaying surgery. Surgeons worried that if they deferred surgery for optimization, the patient would simply find another surgeon who was willing to operate. In addition, surgeons worried that patients would be averse to engaging in challenging health behavior change before surgery. Therefore, despite recognizing the benefits of optimization, surgeons were nevertheless willing to perform surgery on high-risk patients for fear of losing referrals and revenue.

**Table.** Dominant Barriers to Practice Change With Representative Quotations

Barrier	Theme	Representative quotations
Financial	Loss of income	“I’d love to say that we’re sticklers about [optimization], but we’re not, you know. In this age and era of patient satisfaction, you send all these patients out and say come back when you quit smoking, they just go find somebody else and then they write you a bad review too, so you have to balance that.”
	Loss of referral	
	Reputational damage	
	Malalignment of reimbursement	“In my practice, we don’t stop for smoking or for diabetes. I mean, we would still offer a repair. I don’t get paid not to operate.”
Knowledge and resources access	Lack of institutional infrastructure	“We don’t really have, you know . . . [an] optimization clinic . . . so I wish we did, because it’s more work on the surgeon’s end. . . . I wish we did.”
	Lack of knowledge about PREP	“So we do have formal weight loss programs run by our hospital, but there’s a disconnect between the outpatient and inpatient realm . . . so I’ll recommend Weight Watchers or a medical weight loss program.”
Practice patterns and organizational barriers	Organizational expectations	“It’s always, for me, about the patient. But as far as outside factors . . . the administration would love us to operate and use the robot, because it’s a great marketing tool.”
	Local practice patterns and expectations	“At the big universities, you can kind of draw the line, but in the communities, I think we’re sort of stuck with that.”
	Clinician autonomy	“I’m a very lazy surgeon. You should do what’s easiest for you. If it would be easy for you, it’s probably easier on the patients.”
		“I don’t read guidelines. I just make it up.”

Abbreviation: PREP, Preoperative Patient Optimization Program.

Second, surgeons attested to a lack of resources to accomplish optimization among high-risk patients. Although a small number of health systems have stand-alone preoperative optimization programs to which surgeons can refer patients, most surgeons did not practice in such a model. Therefore, any work to improve a patient’s health before surgery fell entirely on the surgeon. This process was seen as burdensome amidst an already busy clinical and operative schedule. Moreover, most surgeons felt that they lacked the expertise in how to effectively counsel patients to improve their health before surgery.

Finally, surgeons identified organizational barriers to deferring surgery until patients had undergone adequate optimization. For example, there was a pervasive belief that health system administration did not view time spent on optimization as high-value patient care. In contrast to already-identified financial barriers in which surgeons raised concerns about losing patient business, surgeons specifically identified expectations to maintain a given volume of operations and asserted that deferral of surgery would decrease this volume.

### Identifying Evidence-Based Implementation Strategies

Using this information obtained from practicing surgeons, the expert task force was convened to deliberate potential implementation strategies to encourage better use of preoperative optimization. Consensus was ultimately reached on 3 implementation strategies to increase surgeon use of preoperative optimization among high-risk patients with hernia.

The first strategy was to conduct educational outreach to address surgeon knowledge gaps and awareness of organizational resources.^31^ This strategy was designed to familiarize surgeons with the readily available, statewide prehabilitation Preoperative Patient Optimization Program (PREP). PREP is a structured prehabilitation program that engages patients in 5 domains: physical activity (patients are provided with a pedometer and encouraged to track steps), healthy diet, breathing exercises (patients are provided with an incentive spirometer), mindfulness, and smoking cessation, if applicable. The details of this program have been described previously.^14,15^ PREP is available to surgeons at MSQC hospitals; however, the previous surgeon interviews demonstrated that most surgeons were unfamiliar with it. Therefore, this implementation strategy involved providing surgeons with training material familiarizing them with the program and instructions to refer patients. Surgeons were also provided with guidelines pertaining to which patients merit referral to PREP (namely, patients with 1 of 3 high-risk characteristics). The overall cost of PREP training and delivery was $7 per referred patient, including the PREP toolkit, surgeon training, and PREP personnel.

The second implementation strategy was active facilitation.^31^ On-site facilitation was developed to encourage surgeon use of preoperative optimization. This strategy was developed based on the recognition that providing passive education alone would likely not achieve the desired outcome at all hospitals. On-site facilitation would provide additional resources to help align the priorities of clinicians and organizational leaders to support adherence to patient optimization. Facilitation is derived from the PARIHS (Promotion Action on Research Implementation in Health Systems) framework, which supports clinicians in the adoption of evidence-based practices through enhancement of strategic thinking and leadership skills that enable them to cultivate surgeon champions to overcome organizational and system barriers to adoption.^32^ For example, in the Recovery-Oriented Collaborative Care trial, on-site facilitation delivered at community care practices in Michigan and Colorado compared with surgeon training alone increased use of a care management program.^33^

The third implementation strategy was to alter the incentive structure surrounding optimization to address financial concerns.^31^ Through an established partnership between Blue Cross Blue Shield of Michigan and the MSQC, a fixed-sum P4P program was developed to financially incentivize surgeons to refer patients for preoperative optimization. Pay for performance is a well-established program already used by the MSQC to incentivize quality improvement efforts.^34^ Under this incentive, hospitals receive a P4P score based on hospital-reported performance regarding preoperative optimization referrals. Hospital payments will then be tiered based on this score, and meeting performance measures enables hospitals to receive an additional fixed 40% financial incentive. Starting in quarter 1 of fiscal year 2021, all hospitals within the MSQC will be eligible for this incentive. Similar financial strategies have been deployed at the statewide level to improve postoperative opioid prescribing.^35^

### Pilot for Improved Preoperative Optimization Using Implementation Strategies

In 2016, PREP training was deployed at 2 sites, including a large academic health system and an affiliate community hospital. These sites were selected based on geographic convenience and willingness of surgeons to participate in pilot implementation efforts. Baseline optimization referral rate was not known for these sites because previous data were deidentified. A subset of surgeons caring for patients with abdominal wall hernia (n = 7) received PREP training. In 2018, referral to PREP increased by 860% from 10 referrals in 2016 to 96 in 2018.

On-site facilitation was implemented in 2018. The on-site facilitator provided clinical guidance and support strategies for high-risk patients, tracked referrals for preoperative optimization, provided feedback, and reinforced guidelines with surgeons. For example, by reviewing medical records for the presence of a high-risk characteristic such as smoking, the facilitator would provide reminders that referral for optimization and even deferral of surgery would be appropriate. Addition of an on-site facilitator was associated with another 40% increase in referral to PREP from 96 referrals in 2018 to 134 referrals in 2019. The incremental total cost of providing the on-site facilitator was $6000 during the 12-month period (0.1 full-time equivalent), which was calculated based on standard salary level for a part-time clinical nurse.

## Discussion

This study describes the development, implementation, and initial results of a multifaceted, stepwise implementation intervention designed to increase surgeon use of evidence-based preoperative optimization for high-risk patients with abdominal wall hernia. Using qualitative interviews with surgeon stakeholders, the primary barriers to evidence-based practice were identified as lack of financial incentive and unfamiliarity with available preoperative optimization programs. Using these results, a P4P model was developed to financially incentivize use of the PREP program, and surgeon training and on-site facilitators provided information and resource knowledge to increase adoption of preoperative optimization. Within the first 2 years of implementing these interventions, the number of patients with abdominal wall hernia referred for preoperative optimization at no cost to participating surgeons increased substantially.

This study demonstrates the use of implementation science methods to overcome widely recognized but poorly addressed barriers to motivating practice change in surgery. Roughly half of the complications that occur after surgery are considered preventable.^36^ Despite abundant evidence regarding the factors that drive these complications and the benefits of preoperative optimization, there is a significant disconnect between published evidence and actual practice.^37^ The bridging of this gap has been shown to depend critically on the methodology of implementation science.^38^ For example, Jafri et al^39^ demonstrated that despite the existence of practice guidelines for the management of abdominal wall hernia in women of childbearing age, wide variation remains in surgeon decision-making. The set of implementation strategies outlined above is an example of an implementation intervention that systematically identifies and overcomes this variation in a stepwise fashion by integrating input from stakeholders. Guided by established implementation frameworks, surgeons identified barriers to optimizing care, which then informed specific strategies to overcome those barriers that can be applied across different sites. To our knowledge, this study is the first to apply these strategies to optimization before a common elective surgical procedure.

In this context, this study also demonstrates the importance of identifying institution- or surgeon-specific barriers to implementation of evidence-based practice. This strategy has been used effectively (eg, when combined with the Theoretical Domains Framework) to map and identify targets for practice change.^19^ In a similar quality improvement initiative, excessive postoperative opioid prescribing was found to arise from surgeon concerns that prescribing less will lead to patient dissatisfaction. By recognizing these beliefs, implementation strategies can be developed that address them, such as behavioral modeling, persuasive communication, and social processes of encouragement.^40^ Insofar as patient engagement in preoperative optimization has been shown to be associated with postoperative outcomes, a similar strategy can be used to assess facilitators and barriers to maximizing participation of patients in optimization efforts. Identifying concerns about financial losses also allowed us to couple these strategies with P4P incentives, which have similarly been used to motivate adoption of evidence-based opioid prescribing practices after surgery.^35^ However, a future state that links P4P to actual preoperative optimization success and not simply referral rate may even further maximize the effect of this intervention.

### Limitations

This study has important limitations. Importantly, the purpose of the present study was to evaluate the design and application of an implementation strategy to bridge a known gap in surgical practice. As such, we did not collect actual outcomes regarding whether optimization referrals led to improvement in risk factors or improved postoperative outcomes. There is a plethora of existing evidence to support the effectiveness of preoperative optimization.^15,41^ This intervention was also conducted within the context of a preexisting collaborative hospital network that greatly facilitated its feasibility. Accordingly, the findings may not be generalizable to other institutions where no such network exists. Nevertheless, similar variation has been demonstrated in other populations of patients undergoing abdominal hernia repair, and similar collaborative hospital networks have emerged to address the challenges of adhering to best practice.^42,43^

Another limitation is the lack of geographic information regarding the distribution of high-risk health behaviors. Smoking, obesity, and unhealthy alcohol consumption are all associated with socioeconomic status, and therefore the case mix and barriers that hospitals encounter will be unique to their geographic setting. Future iterations of this work should address these variations in local practice so that solutions tailored to a given hospital’s patient population can be implemented. In addition, to the extent that other health care systems and surgical practices may have different barriers to adoption of evidence-based practice, the strategy described herein is generalizable in that it would enable those systems to engage stakeholders in identifying their own unique barriers and design implementation strategies accordingly.

Another limitation is that the overall proportion of high-risk patients was not tracked after implementation of this intervention strategy, so the extent to which additional high-risk patients were not being referred for optimization is unknown. It is similarly unknown how much improvement was due to the addition of an on-site facilitator vs growing awareness of optimization among surgeons. Although we have no reason to believe the patient population at these sites changes substantially during the study period, future efforts should track all patients such that changes in referral rates can be understood in a larger context. Specifically, our intervention did not make use of any automated referral technology or real-time decision support tools, which have been well proven to increase the efficiency and sustainability of similar quality initiatives. There are also limitations in using theoretical domains framework, including the lack of a formal way in which to apply the framework in the health care setting.^20^

## Conclusions

This quality improvement study illustrates a multifaceted, stepwise process to develop an implementation intervention aimed at overcoming barriers to the adoption of evidence-based optimization of patients with abdominal wall hernia. This process used a systematic method of identifying variation in real-world clinical practice, recognizing the drivers of this variation through in-depth interviews with surgeon stakeholders, developing interventions to reduce variation, and ultimately piloting these interventions. Results from this study will inform larger-scale quality improvement efforts to increase the use of evidence-based practice for patients with abdominal wall hernia at a statewide level.
